# Qualitative inquiry of cancer caregiving during young adulthood: responsibilities, challenges, teamwork, and social support

**DOI:** 10.1097/or9.0000000000000062

**Published:** 2021-10-25

**Authors:** Austin R. Waters, Lisa H. Gren, Charles R. Rogers, Anne C. Kirchhoff, Echo L. Warner

**Affiliations:** a Cancer Control and Population Sciences Research Program, Huntsman Cancer Institute, Salt Lake City, UT,; b Division of Public Health, Department of Family & Preventive Medicine, University of Utah School of Medicine, Salt Lake City, UT,; c Department of Pediatrics, University of Utah, Salt Lake City, UT,; d University of Arizona Cancer Center, Tucson, AZ,; e College of Nursing, University of Arizona, Tucson, AZ.

**Keywords:** cancer caregiving, social support, young adult

## Abstract

**Background:**

Young adult cancer caregivers (YACC) may experience heightened caregiver burden because they take on caregiving during a dynamic time of life. The purpose of this study was to describe YACC experiences, burden, and social support while caregiving.

**Methods:**

Grounded by the Cancer Family Caregiving Experience Model and the Stress and Coping Social Support theory, semi-structured interviews were conducted with YACC. Inductive analysis was applied to YACC perceptions of caregiving, and deductive analysis to YACC social networks and social support.

**Results:**

YACC (N=34) were primarily between 25 to 29 years of age (38.2%), primarily female (70.6%), non-Hispanic White (91.2%), employed (85.3%), college graduates or higher (53.0%), and caring for a spouse/partner (52.9%). Qualitative analysis of interviews with YACC resulted in 2 themes: cancer caregiving during young adulthood and young adult cancer caregiver social support. In the first theme YACC often did not perceive themselves as caregivers, rather their caregiving responsibilities were viewed as an extension of their relationship with the patient. Further YACC reported developmental-specific responsibilities (eg, caring for young children, being unable to take time off while solidifying careers) which often conflicted with their caregiving responsibilities (eg, managing patient’s medication, attending appointments) and heightened burden. In response, YACC often formed caregiver teams consisting of family, friends, and community members to care for their loved one. In the second theme YACC most commonly reported receiving emotional and instrumental support from their social network. YACC specifically mentioned their appreciation for emotional support provided by other young adults and instrumental support in the form of financial support.

**Conclusions:**

YACC faced developmentally specific challenges during caregiving that older adult caregivers may not encounter. The conflict of young adult and caregiving responsibilities resulted in YACC forming caregiver teams. Thus, theoretical approaches to understand and improve caregiver health would benefit from the inclusion of the developmental context of young adulthood. Furthermore, it is crucial that cancer centers tailor supportive services to YACC as the number of young caregivers increases.

## Background

Adults 65 years and older are the fastest growing age group in the United States,^[1]^ and are diagnosed with over half of all cancers.^[2]^ Family members often take on a caregiver role by providing unpaid care to a cancer patient.^[3]^ This care can take many forms but commonly caring directly for the patient includes symptom management, treatment and medication monitoring, and emotional support.^[4,5]^ Caregiving also encompasses everyday tasks such as cooking, cleaning, and grocery shopping that can no longer be accomplished by the patient due to their diagnosis.^[4,5]^ Although caregiving for a loved one with any serious health condition can be burdensome, cancer caregiving presents unique burden,^[3]^ resulting from high levels of care required over long periods of time and expensive treatments. These burdens are negatively associated with cancer caregiver social, emotional, and financial well-being.^[6,7]^

Due to shifting demographics, young adults increasingly engage in caregiving roles.^[8,9]^ Of the projected 6.5 million caregivers for older adults with a serious health condition in the United States, roughly 25% are aged 18 to 34 years.^[10]^ Beyond young adult caregivers increased involvement with caring for older adults they may have a variety of relationships with the patient including spouse, friend, or sibling. Young adults are particularly vulnerable to negative caregiving outcomes as they take on caregiving roles during the dynamic transition of young adulthood.^[3,11]^ According to Arnett (1998, 2000), cognitive individualism and the transition to stability are hallmarks of young adulthood, including: taking responsibility for one’s self; independent decision making; intimate relationships development; forming families and having children; solidifying careers and establishing financial independence.^[11,12]^ Thus, young adult cancer caregivers (YACC) may struggle to balance the transitions of young adulthood with the responsibilities of caregiving for a cancer patient.

Cancer caregivers often experience high rates of social isolation, anxiety, depression, and low quality of life (QOL).^[13–15]^ The negative impacts of cancer caregiving may be amplified for YACC when the responsibilities of cancer caregiving overlay, and conflict with, young adulthood development.^[8,16]^ Although literature on how cancer caregiving impacts YACC remains limited, robust social support is positively associated with better health outcomes among cancer caregivers of all ages.^[17,18]^

Social support moderated the impact of psychological distress on QOL among spousal cancer caregivers during childbearing years,^[19]^ and mediated the distress from cancer-related stressors for older caregivers.^[18]^ Additionally, cancer caregiver social support was associated with better lung cancer patient health,^[17]^ highlighting the complex and interdependent nature of patient and caregiver support and health. Social support may be particularly important for YACC as social relationships are an important developmental aspect of young adulthood.^[12]^ Thus, social relationships and support may contribute to YACC wellbeing differently than observed for older caregivers.

However, information about YACC social relationships, young adult specific caregiver burden, and social support are limited.^[8,20]^ Accordingly, we analyzed data from semistructured interviews with YACC to describe their perceptions of their cancer caregiver role and caregiver burden as a young adult, and identify who provides them with social support while caregiving. Understanding how YACC perceive and experience caregiving may inform supportive services for young adults as they increasingly take on cancer caregiving roles.

## Methods

### Theoretical foundation

The Cancer Family Caregiving Experience Model (CFCEM) informed this study.^[21]^ The Cancer Family Caregiving Experience Model (CFCEM) was used to contextualize caregiver burden and stress described by YACC in this study. CFCEM describes the caregiver stress process wherein caregivers experience primary stressors (eg, caregiving responsibilities) and secondary stressors (eg, relationship strain, financial outcomes) which trigger cognitive appraisal, where YACC assessment of their stressors results in a cognitive behavior response (eg, coping, planning ahead).^[21]^ Cognitive appraisal is embedded in contextual factors such as demographics and social support.^[21]^ The Stress and Coping Social Support Theory also informed this study due to its foundational hypothesis that the individual’s perception of their social support impacts the appraisal process which mediates the impact of a stressor on health.^[22]^ Furthermore, this theory was used during analysis to categorize social support received by YACC into 5 distinct types: emotional, instrumental, validation, companionship, and informational (Table 1 for definitions).

### Participants and recruitment

This qualitative study was part of a larger mixed-methods study on YACC use of social media that collected information on types of, and resources for, support and change in support over the first 6 months of cancer caregiving. Eligibility criteria included individuals who were: age 18 to 39 years, a recent cancer caregiver (ie, provided unpaid care to an adult cancer patient for a minimum of 6months up to 5 years before data collection), using social media regularly (at least once per week), and speaking English. Participants were recruited online through fliers and social media shared by cancer/cancer caregiving organizations, as well as in person through the Huntsman Intermountain Adolescent and Young Adult Cancer Care Program. Health care providers and research staff affiliated with Huntsman Cancer Institute and Intermountain Healthcare also referred patients with an eligible caregiver. Patients were screened by study staff and asked to refer their primary caregiver. Although the parent study primarily focused on social support obtained through social media,^[20]^ our research team examined and reports here non–social media-based social support. All study procedures were approved by the University of Utah Institutional Review Board (IRB #00097575).

### Data collection and qualitative analysis

Interview questions were either open ended to explore emergent topics regarding YACCs’ perceptions of caregiving as a young adult or structured to identify the type of functional social support provided to YACC and by whom. Thus, open-ended questions were analyzed using an inductive phenomenological approach to focus on finding shared meaning among participants’ lived experiences.^[23]^ Guided by the Stress and Coping Social Support Theory,^[22]^ a deductive content analysis approach was employed to analyze the structured questions about the types and sources of participants’ perceived social support.^[24]^ This theory was also used to categorize support provided to YACC into the 5 functional social support types (Table 1) by individuals who provided it. Examples of semistructured interview questions and their corresponding analytic approaches (ie, inductive vs deductive) are provided in Table 2. Interviews were recorded, transcribed, and quality checked against audio files to assure accuracy. Due to the different types of questions asked, YACC responses to open- and close-ended interview questions differed substantially, warranting different analytic approaches (ie, open-ended questions and inductive analysis; close-ended questions, and deductive analysis). Sociodemographic information collected at the beginning of each interview was stored in REDCap, and included: age, gender identity, race, ethnicity, sexual orientation, employment status, highest level of education obtained, relationship to patient, and whether they were caring for another individual. All data were analyzed via 2 rounds of structured coding^[25]^ using NVivo 11 (QSR International, Melbourne, Australia).

First cycle coding was applied by the lead author (ARW) to 10% of interviews to categorize data based on emergent ideas.^[25]^ During second cycle coding, a further 10% of interviews were coded by 2 members of the research team (ARW and ELW). Discrepant codes were resolved through discussion of coding rules, resulting in coding scheme refinement. An additional 10% of interviews were double coded until intercoder reliability met the threshold for high reliability (inductive κ=.831; deductive κ=.834).^[26]^ The final coding scheme was applied to all 34 interviews. To sort codes and describe differences in experiences and social support by developmental age, transcripts were marked by relationship to patient (eg, spouse, sibling, child) and age (younger: 18–25 vs older: 26–39).

All interviews were conducted by ELW, who was a PhD candidate in Nursing during data collection. ELW is a first-generation college student from rural Utah and had 7 years of research experience with AYA cancer caregivers and patients. Analyses were conducted by ARW and ELW. ARW is a queer male and was a public health graduate student and research program manager for an AYA cancer researcher at Huntsman Cancer Institute during data collection and analysis. ARW has personally been a young adult caregiver for both blood and chosen family members.

## Results

**FIGURE 1.**
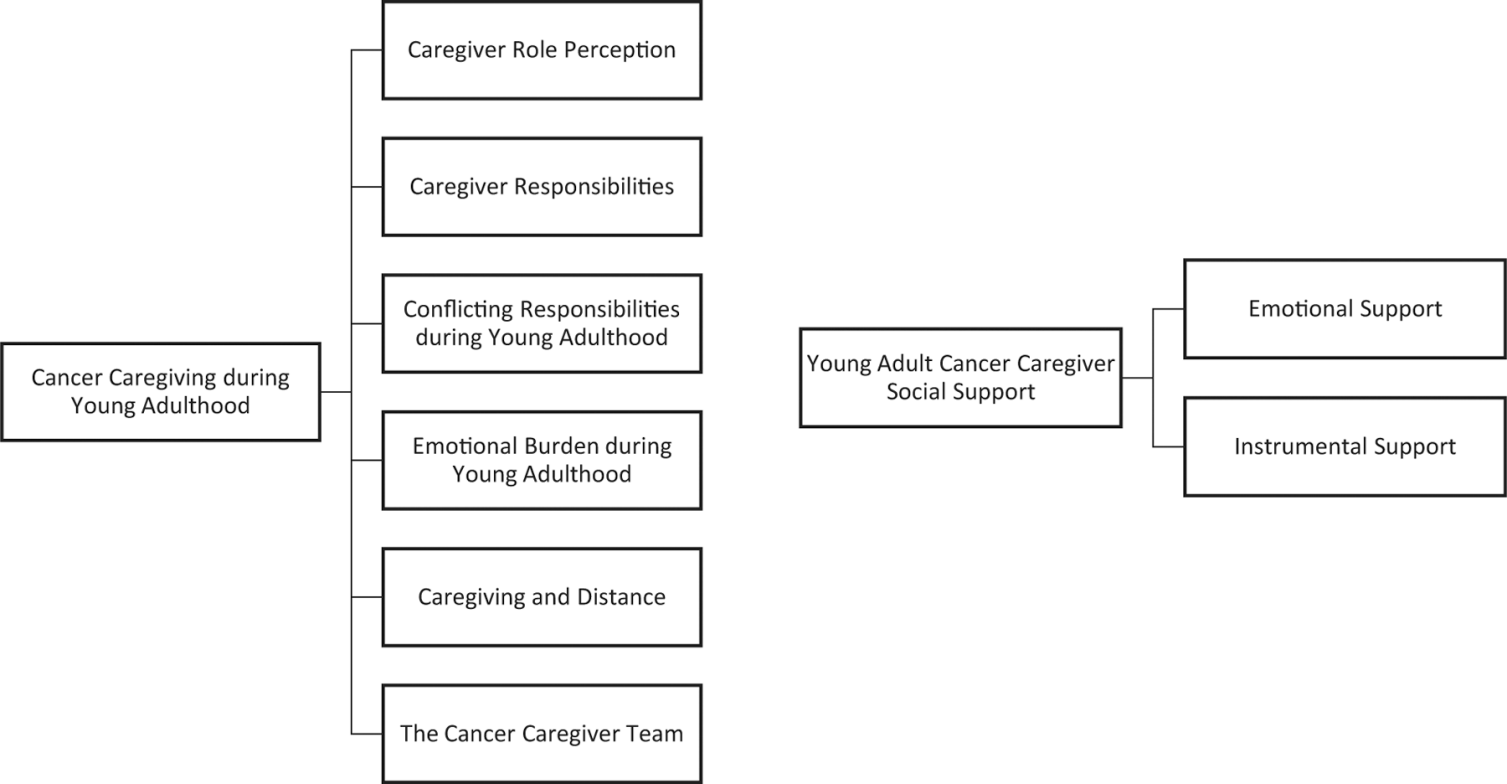
Categories and subcategories of young adult cancer caregivers’ caregiving experiences and social support.

Of 354 cancer patients screened for the parent study, 48 eligible young caregivers were identified; 8 declined and 6 were lost to follow up. The remaining 34 provided informed consent (70.8% participation rate) and participated in a telephone-based semi-structured interview, lasting between 41 and 79 minutes. YACC were most often aged 25 to 29 (38.2%), female (70.6%), non-Hispanic White (91.2%), heterosexual (97.1%), employed (85.3%), college graduates or higher (53.0%), and caring for a spouse or partner (52.9%) or parent (23.5%) (Table 3). Qualitative analysis resulted in 2 distinct categories: cancer caregiving during young adulthood and young adult cancer caregiver social support (Fig. 1).

### Cancer caregiving during young adulthood

The 6 subcategories pertaining to cancer caregiving during young adulthood are discussed below (Fig. 1). Although YACC commonly did not identify with the “cancer caregiver” title (subcategory 1) they performed a variety of caregiver responsibilities (subcategory 2). The difficulties experienced while caregiving were exacerbated by developmental context as described in subcategories 3 to 5: conflicting responsibilities during young adulthood, emotional burden during young adulthood, and caregiving and distance. These difficulties resulted in the formation of caregiver teams which YACC leveraged to care for the patient (subcategory 6). Illustrative quotes for each subcategory are found in Table 4.

#### Caregiver role perception.

All YACCs viewed themselves as providing some sort of assistance to their loved one with cancer, although there were differences in identifying with the caregiver role. Over two-thirds of YACC did not view themselves as cancer caregivers, rather they reported performing caregiving activities because they viewed these activities as a natural extension of their relationship. However, YACC who did identify as a cancer caregiver emphasized the role was defined by added responsibility to the cancer patient. These caregivers often viewed health as multidimensional and reported that caregiving included supporting the patient’s psychosocial and physical health. Role perception was not reliant on the type of relationship the YACC had with the patient nor their age.

#### Caregiving responsibilities.

YACC most often engaged in caregiving responsibilities that related to the cancer treatment of the patient, including: attending appointments; refilling, administering, and tracking medications; managing side effects and recovery; and medical decision making. Additionally, the psychological toll of cancer on the patient often led YACC to support the patient emotionally, including: helping them stay positive; maintaining a sense of normalcy; and being there for the patient to talk to. YACC also often took on daily living responsibilities, including: cleaning and upkeep of the patient’s household; preparing meals; assisting financially; and even working the patient’s job (eg, farming, running a small business). YACC who were taking care of a young adult patient reported additional developmentally relevant tasks including: filing university leave of absence or scholarship deferral forms; and providing childcare for the patient’s children.

#### Conflicting responsibilities during young adulthood.

Most YACC reported that the added responsibilities of caregiving were layered on top of existing young adult responsibilities and they frequently felt they did not have enough time to fulfill both roles’ responsibilities. In addition to caring for the patient, these responsibilities included: dating or forming meaningful relationships; working full-time and/or pursuing higher education; and taking care of young children (their own or the patient’s). Caring for young children was particularly problematic, as some caregiving responsibilities precluded children, such as attending appointments where children were not allowed.

#### Emotional burden during young adulthood.

Caregiver burden was heightened by the emotional impacts of caregiving including a sense of uncertainty about how to best care for the patient. Their inexperience in caring for someone with a serious illness caused YACC to feel initially overwhelmed by the diagnosis, their caregiving responsibilities, and managing support that was offered by their social network. At the same time, taking on a caregiver role at such a young age also challenged some YACC perspectives of invincibility, which complicated the emotional stress caused by conflicting responsibilities and roles. In addition, YACC expressed a need for their opinions to be heard and valued during caregiving, which at times led to frustration, conflict, and strained relations between family members sharing caregiving responsibilities.

#### Caregiving and distance.

Although much less common than burden due to young adult responsibilities or limited caregiving experience, distance from the patient and hospital substantially increased burden for certain caregivers. A few YACC reported regularly traveling up to 300 miles to pick up the patient and attend appointments. Long distance travel was particularly burdensome as YACC found themselves with less time for their jobs, school, and families because of time spent transporting the patient to appointments.

#### The cancer caregiving team.

Constraints of time and emotional stress due to conflicting responsibilities of YACC resulted in the formation of caregiver teams. Caregiver teams consisted of individuals from YACC and patients’ social networks (eg, family members, friends, and community members). Formation of such teams was based on pre-existing relationships, as well as unique strengths of team members. For example, close family members or friends frequently provided emotional support and helped take care of children, while those with medical training—regardless of how close they were to the patient—played a significant role in bridging health literacy gaps and helping with treatment decisions. YACC frequently mentioned non-family members (eg, friends, co-workers) taking major roles in their caregiver teams; this was especially true for younger YACC. For example, some younger YACC reported their friends providing direct support to the patient when they were unavailable. YACC commonly spoke about their caregiving experience using plural rather than singular pronouns (eg, we vs I), focusing almost exclusively on caregiving in the team context rather than individual effort.

### Young adult cancer caregiver social support

This category emerged from deductive qualitative content analysis and included 2 subcategories (Fig. 1). Although coding occurred for all 5 types of functional social support, as described in the Stress and Coping Social Support Theory, YACC rarely reported receiving social support in the form of companionship, validation, or information support; rather, they primarily reported emotional and instrumental support. Illustrative quotes for each subcategory can be found in Table 5.

#### Emotional support.

YACC reported receiving emotional support from a variety of individuals (eg, coworkers, neighbors, church members), but mostly family and friends. YACC felt emotionally supported when individuals checked in on how they were doing and when they could express their caregiving frustrations. Some YACC viewed friends as a safe place to vent without upsetting the dynamics between those helping with caregiving tasks. YACC commonly talked with close family about their feelings but also valued more detached conversations with their friends. The positive impacts of these conversations were particularly noted for YACC taking care of their spouse. YACC were particularly grateful for emotional support provided to them by individuals who understood their feelings and perspectives as a young adult.

#### Instrumental support.

Instrumental support included: preparing meals; childcare; cleaning during immunocompromising treatments; help with medical decision making; transportation; and financially supporting YACC or the patient. YACC reported instrumental support primarily from family and friends, with some additional support from community members or neighbors. With the exception of medical decision making (primarily handled by close family or with input from medical professionals who were not close family), provision of instrumental support was not dependent on relationship to YACC. Older YACC received more instrumental support from family than friends, whereas younger YACC received nearly equal amounts of instrumental support from family and friends. Although YACC felt grateful for all of the instrumental support they received, they emphasized the importance of financial support.

## Discussion

In addition to navigating the challenging time of young adulthood, YACC often face sizeable emotional burden and conflicting responsibilities that lead to the formation of caregiver teams. Consistent with emerging literature,^[27]^ YACC in our study reported forming caregiver teams, which typically included other family members and friends whose roles were determined by individuals’ strengths. Although most cancer caregiver literature focuses on patient-caregiver dyads,^[3,17,21]^ our results suggest YACC may benefit from future research and supportive services expanding past the dyad and focused on larger, more comprehensive social support networks instead. Due to the non-dyadic nature of caregiving teams, YACC friends commonly played substantial roles in caring for both the YACC and the patient. This finding is consistent with literature on young adult development about increased reliance on friendships during young adulthood, specifically when unmarried,^[12,28]^ and illustrates how deeply influenced cancer caregiving is by developmental age.

Our findings that YACC commonly form caregiver teams diverges from the dyadic focus of the Cancer Family Caregiving Experience Model (CFCEM), the most current cancer caregiver theory.^[21]^ Our findings imply YACC face primary stressors similar to older adults^[5]^ as they perform largely the same caregiving tasks. However, YACC face young adult-specific stressors and context (ie, developmental age) that are not fully encapsulated by the CFCEM’s description of secondary stressors as “spillover effects.” This finding suggests that the CFCEM could benefit from expanding the model to include how contextual factors can also act as stressors and the potential impact these contextual stressors may have on the rest of the stress process. Our findings also suggest that YACC form caregiver teams when they encounter an “overflow point,” at which they experience an overwhelming amount of primary, secondary, and contextual stressors. When cognitively appraised, this “overflow point” led YACC in our study to no longer view their caregiving as feasibly dyadic and resulted in the formation of teams as a cognitive behavioral response. This description of YACC caregiver teams is novel in that previous dyadic caregiver research inferred that caregivers have the ability to assess their stressors and cope with them dyadically with the patient.^[3,17,21]^ Yet, this may not fully describe the experiences of caregivers with contextual factors, such as developmental age, that act as stressors. Future research informing the CFCEM should assess whether social support provided by a caregiving team is sufficient for mitigating the added contextual stressors associated with developmental age, in addition to whether caregiver team stress processes, social support, and health are interdependent, as is seen in patient-caregiver dyads.^[17,21]^ Further, future research should assess the influence of caregiving teams on the documented positive aspects of cancer caregiving such as forming deeper relationships with the patient as well as caregiver personal growth and satisfaction.^[29]^

Irrespective of their caregiver team participation, our YACC participated in a substantial amount of caregiving, performing nearly equivalent tasks as older caregivers.^[5]^ Similar to findings from a study by Shaw et al with a broad age-range of caregivers,^[30]^ YACC often did not view themselves as cancer caregivers; rather, they saw caregiving as an extension of their existing relationship with the patient. While caregiving, YACC often reported emotional burden as they experienced uncertainty of best caring for their patient. Consistent with reports in older cancer caregivers, our findings indicate that lack of preparedness for the caregiving role is associated with higher levels of anxiety, depression, and guilt.^[31]^ Consistent with literature on characteristics of young adult development,^[9]^ our YACC reported emotional burden due to dealing with mortality at a non-normative time of life and their need for their opinions to be heard during caregiving. Hence, YACC may experience different caregiver emotional burdens and may not identify with the available cancer caregiver supportive services due to their role perception. This finding is concerning, as mental health support services are consistently identified as an unmet need for cancer caregivers of all ages.^[32]^ Future research should further explore supportive services in modalities and cover topics targeted at addressing the challenges of caregiving during young adulthood. Specifically, YACC emotional health may benefit from supportive services that help them cope with non-normative developmental tasks such as grappling with mortality during young adulthood.

In addition to potential negative emotional health impacts of caregiving as a young adult, our results suggest developmental-specific life responsibilities (eg, taking care of young children, working full-time, going to school) may add to the challenge of caregiving for YACC. Specifically, financial stability, YACC often reported feeling very thankful for the needed instrumental financial support, which is consistent with literature on financial toxicity and cancer treatment unaffordability.^[33]^ Since financial toxicity is heightened among young adults,^[34]^ it may negatively impact wealth and economic mobility of both caregivers and patients. Furthermore, consistent with our findings concerning travel burden to fulfill caregiving responsibilities, young adults are more likely to move away from family for developmental opportunities (eg, higher education, jobs, starting a family).^[35]^ These findings suggest future research to mitigate the negative impacts of financial toxicity (eg, job lock, bankruptcy) should consider developmental age.

### Clinical implications

Similar to supporting cancer caregivers of any age, fully supporting YACC may require substantial investment from cancer centers to systematically assess caregiver burden and health.^[3]^ Cancer care teams can engage YACC as part of the medical care team while acknowledging that YACC may be less prepared to provide care to the patient than older caregivers and may engage others in caring for the patient. In comparison to older adults, young adults in general are much more diverse and may have different caregiving and support network structures than what cancer care providers have previously encountered (eg, the growing number of LGBTQ_+_ caregivers and the chosen family that support them).^[36,37]^ Furthermore, YACC may seem less engaged in caregiving due to conflicting responsibilities but should not be mistaken for providing less support to the patient. When engaging with YACC cancer care providers should frame YACCs’ participation in caregiving within their developmental context and conflicting responsibilities. Lastly, due to YACC often being new to navigating health systems and helping with medical decision making, they may be susceptible to misinformation. Cancer care providers can help mitigate some YACC stress and exposure to misinformation by recommending trustworthy sources of medical information and being one themselves, particularly online.^[38,39]^

### Strengths and limitations

Our description of YACC caregiving experiences is a unique contribution to the literature that can inform future research on caregiver theory and targeted YACC interventions. Two limitations are relevant. First the perspectives of non-Hispanic white, heterosexual, highly educated, spousal caregivers were overrepresented due to our sample demographics and recruitment strategies. Although the perspectives in this study may be limited by sample demographics, describing cancer caregiving in the context of developmental age is novel. Since minority caregivers already experience unique challenges,^[40,41]^ and young adult demographics in the United States continue to diversify,^[9]^ future research should explore developmentally specific challenges in YACC from diverse racial, ethnic, and/or sexual minority demographics. Additionally, since our exploratory analysis did not reveal an impact of patient relationship on caregiving perceptions, further research should explore whether relationship modifies perceptions of developmentally-specific challenges in YACC. Secondly, YACCs were not asked to categorize their support, and thus companionship, informational, and validation support—as categorized by coders—were not frequently reported. This may be a result of coding, or simply that YACC did not identify with this type of support, yet our internal consistency was highly reliable (k=.83).

## Conclusions

Cancer caregiving during young adulthood poses developmentally specific challenges that may not be encountered by older adult caregivers. Experiencing conflicting young adult life responsibilities, YACCs are often ill-equipped to take on a caregiver role and have their young adult perspectives challenged while caregiving. These factors may heighten YACC emotional burden. Furthermore, YACC often do not perceive themselves as cancer caregivers, which may limit their use of supportive services. Caregiver supportive services may benefit from targeting YACC without using the title “cancer caregiver” while concurrently providing services developmentally tailored and delivered through modalities used by young adults. Due to the layering of caregiving responsibilities and young adult responsibilities, YACC often reported forming caregiver teams. YACC caregiving in a non-dyadic manner implies an opportunity for existing theoretical models to expand their frameworks to incorporate how contextual factors such as developmental age can act as a stressor, thus impacting caregiver stress processing, social support, and health. Future research should focus on further describing how YACC process stress and whether the formation of caregiver teams is adequate to mitigate developmentally specific stressors, as well as whether there are differences based on the relationship to the patient. Tailoring and targeting social support services to YACC is of the upmost importance as the frequency of young adults taking on cancer caregiver roles will only continue to rise as the US population ages.

## Figures and Tables

**Table 1:** Stress and Coping Social Support Theory definitions of functional social support

Functional social support	Definition
Emotional	Sympathy, caring, acceptance
Instrumental	Transportation, household chores, child-care, finance
Informational	Knowledge, information, advice, alternative action
Companionship	Availability of persons to spend time with
Validation	Feedback, social comparison

**Table 2:** Examples of Semi-Structured Interview Questions and their corresponding analytic approach

Example interview questions	Analytic approach
Some people don’t really think of themselves as cancer caregivers even though they are taking care of someone with cancer. What do you think about this? Probe: What has your experience been like taking care of someone with cancer?	Inductive analysis
Who are the people in your life who give you support? These people might be near to you or far away. Probe: Who gives you support when you are taking care of your loved one with cancer? You might get support from someone you know personally, someone you know online or even your close coworkers. Probe: What kind of help do these people provide to you? Probe: How has this help changed since your loved one was diagnosed with cancer until now?	Deductive analysis

**Table 3:** Young adult cancer caregiver sociodemographics (N=34)

	N	%
Age at interview		
18–24	4	11.8
25–29	13	38.2
30–34	9	26.5
35–39	8	23.5
Gender		
Female	24	70.6
Male	10	29.4
Race*		
White	31	91.2
African American	1	2.9
American Indian or Alaska Native	2	5.9
I don’t know	1	2.9
Ethnicity		
Hispanic or Latino	3	8.8
Non-Hispanic nor Latino	31	91.2
Education		
High school or less	3	8.8
Some college	13	38.2
College graduate or higher	18	53.0
Sexual orientation		
Heterosexual	33	97.1
Other	1	2.9
Caregiver’s relationship to patient		
Spouse/partner	18	53.0
Child	8	23.5
Sibling	5	14.7
Other (eg, cousin)	3	8.8
Caring for others besides the patient	23	67.6

* Percentages add up to >100% as Young Adult Cancer Caregiver were able to select >1 option.

**Table 4:** Illustrative quotes for the category “Cancer Caregiving during Young Adulthood” and associated subcategories.

Subcategory	Illustrative Quotes
Caregiver role perception	I don’t consider myself as a cancer caregiver. I just consider like I am taking care of my sister because I love her and she is my sister. (30–34 year old female caring for sibling) I was doing most of the things, I took over a lot of his responsibilities in order to help him, I guess is how I know I was a caregiver. (25–29 year old female caring for spouse/partner) When I heard the name of the study I had the same reaction. I don’t feel like a caregiver, as in that’s her medical team or to the extent that she needs immediate physical needs met. (35–39 year old female caring for sibling)
Caregiver responsibilities	she doesn’t have energy to do housework, so we do all the cooking, all the grocery shopping, all the cleaning, all the yard work, all the running of the errands, you know? (25–29 year old female caring for parent) And being for the most part, I for a lot of it managed all his medications, took care of getting refills, got a pill reminder, put them in there, set reminders on his phone to take them. (25–29 year old female caring for spouse/partner) Trying to pick him up and take him places to help his mood or just like get him out of the house a little bit, take him out to see the cows or whatever, that’s easy to do that he’ll enjoy. (25–29 year old female caring for parent)
Conflicting responsibilities during young adulthood	I still attend her doctor appointments if I have time, like if it’s not a conflict of my work schedule. (35–39 year old female caring for parent) Yeah, so we had to just kind of, at the time we had a two month old baby and a four year old and a two year old. So we had to leave them, hurry up here [cancer center], then it was just kind of a whirlwind. (25–29 year old female caring for spouse/partner) I’m like oh my gosh, how am I going to be able to cut myself into five different people here, and be five different places all at once? (30–34 year old female caring for sibling) It’s not just me, because I have a full-time job unfortunately. It’s reality. I wish I didn’t have to work, but I do have to work. So, we kind of all take turns in helping her as much as we can. (30–34 year old female caring for sibling)
Psychological burden during young adulthood	I didn’t quite know how to take care of my wife with her being like a child [very sick during treatment] and being so kind of helpless which I think was so shocking and scary to me. (25–29 year old male caring for spouse/partner) I mean, you come to the realization of life that you’re not unstoppable. (30–34 year old male caring for spouse/partner) I remember there was kind of a space in time where I’m like we are going to rip each other’s heads off. (35–39 year old female caring for other)
Caregiving and distance	We live in [city outside of Utah], so it’s about a three-hour drive one way. So, it kinda added up, quite a bit of time off and driving and expense. (35–39 year old male caring for spouse/partner) And I work in a different state. And so, I usually have to travel there, once a week, at least. And so, adjusting that schedule, I was going to Nevada less and staying here more. (25–29 year old female caring for parent)
The cancer caregiver team	I mean, we are really lucky to have a big support group of family and friends that were able to watch our kids when we had to leave at the last second, when things change. (25–29 year old female caring for spouse/partner) It was nice that she knew what questions to ask. Like she would ask when I told her, she was like what kind is it? But it was also what kind is it, what stage is she at, how is she doing, what treatment are they going to go with? (25–29 year old male caring for parent) Everyone helps to lift or carry the burden, if you will. (35–39 year old female caring for sibling) And so, we’ve kinda all just had to get together and support each other. (35–39 year old male caring for spouse/partner) Definitely. And our group chat, I just think “team [patient initials]. (35–39 year old female caring for sibling) It, it just wouldn’t be possible, it wouldn’t have happened without my family, my parents and brothers and sisters, and aunts and uncles, and cousins and grandparents, friends from school. (25–29 year old female caring for spouse/partner)

**Table 5:** Illustrative quotes for the category “The Caregiving Team and Social Support” and associated subcategories.

Subcategory	Illustrative quotes
Emotional support	And then also she’s a support system for me, just to talk about what’s going on and she’s an outside person that I can talk to that’s not like kinda in the mix of it, to where I’m not venting to the wrong person. (35–39 year old female caring for parent) And then like the best like person to talk to, I think, was probably my brother because you know I think well, I could talk to my parents about it and my grandparents, whatever, and it was always comforting and couldn’t have done without them, but probably the actually the most help I ever got was from my brother. (18–24 year old male caring for sibling) My sister and I have two really good cousins that we talk to and we hang out with. (30–34 year old female caring for sibling)
Instrumental support	We’ve had financial support as well, which is huge, we’ve needed that. (25–29 year old female caring for spouse/partner) He helps out you now, with the kids, because if I have to go to a doctor appointment for my mom. He’ll leave and take on the kids. (35–39 year old female caring for parent) Like hey do you, sometimes they would bring dinner if I didn’t have time to cook dinner if I didn’t have time to cook dinner, or take us out to dinner. Like don’t cook. Don’t worry about it. C’mon let’s go gets come food. (30–34 year old female caring for sibling)
